# Down-regulation of microglial activity attenuates axotomized nigral dopaminergic neuronal cell loss

**DOI:** 10.1186/1471-2202-14-112

**Published:** 2013-10-04

**Authors:** Dae-Yong Song, Ha-Nul Yu, Chae-Ri Park, Jin-Sook Lee, Ji-Yong Lee, Byung-Gu Park, Ran-Sook Woo, Jung-Tae Han, Byung-Pil Cho, Tai-Kyoung Baik

**Affiliations:** 1 Department of Anatomy and Neuroscience, Eulji University School of Medicine, 143-5, Yongdu-dong, 301-832 Jung-gu, Daejeon, Republic of KoreaKorea; 2 Department of Anatomy and Institute of Lifestyle Medicine, Yonsei University Wonju College of Medicine, Wonju, Republic of Korea

**Keywords:** Medial forebrain bundle (MFB), Axotomy, Activated microglia, Reactive oxygen species, Tuftsin fragment 1-3

## Abstract

**Background:**

There is growing evidence that inflammatory processes of activated microglia could play an important role in the progression of nerve cell damage in neurodegenerative disorders such as Parkinson’s disease and Alzheimer’s disease which harbor features of chronic microglial activation, though the precise mechanism is unknown. In this study, we presented *in vivo* and *ex vivo* experimental evidences indicating that activated microglia could exacerbate the survival of axotomized dopaminergic neurons and that appropriate inactivation of microglia could be neuroprotective.

**Results:**

The transection of medial forebrain bundle (MFB) of a rat induced loss of dopaminergic neurons in a time-dependent manner and accompanied with microglial activation. Along with microglial activation, production of reactive oxygen species (ROS) was upregulated and TH/OX6/hydroethidine triple-immunofluorescence showed that the microglia mainly produced ROS. When the activated microglial cells that were isolated from the substantia nigra of the MFB axotomized animal, were transplanted into the substantia nigra of which MFB had been transected at 7 days ago, the survival rate of axotomized dopaminergic neurons was significantly reduced as compared with sham control. Meanwhile, when the microglial activation was attenuated by administration of tuftsin fragment 1-3 (microglia inhibitory factor) into the lateral ventricle using mini-osmotic pump, the survival rate of axotomized dopaminergic neurons was increased.

**Conclusion:**

The present study suggests that activated microglia could actively produce and secrete unfavorable toxic substances, such as ROS, which could accelerate dopaminergic neuronal cell loss. So, well-controlled blockade of microglial activation might be neuroprotective in some neuropathological conditions.

## Background

Microglia, the primary immune effector cells in the central nervous system (CNS), are distributed throughout the CNS and constitute approximately 10-20% of the total glial cell population within the brain [1]. Under physiological conditions, microglia are important in maintenance and restoration of the brain by scavenging the invading infectious agents and any by removing foreign materials, dying cells, or random cellular debris. However, if microglia become activated in response to injuries to the brain or to immunologic stimuli, they undergo morphological changes, proliferate, and increase the expression of major histocompatibility (MHC) complex molecules and complement receptor type 3. Activated microglia also release a variety of proinflammatory soluble factors [2-5]. Microglial activation is a histopathological hallmark of several neurodegenerative disorders, including Parkinson’s disease, Alzheimer’s disease, multiple sclerosis, and the AIDS dementia complex [6-9]. Over the last ten years, there has been an increased interest in microglia because microglial activation could be a risk factor triggering a cascade of events leading to progressive neuronal degeneration [10,11].

Parkinson’s disease (PD) is a neurodegenerative disorder characterized by progressive loss of dopaminergic (DA) neurons in the substantia nigra pars compacta (SNpc). Although the cause of DA neuronal death in PD remains unclear, accumulating evidences strongly suggest that microglial activation and accompanying neuroinflammation may be one of the factors contributing to the pathogenesis of PD. For example, the accumulation of activated microglia and up-regulation of several inflammatory cytokines has been observed in association with degeneration of DA neurons in the substantia nigra (SN) of postmortem PD brain and animal models of PD treated with the administration of 1-methyl-4-phenyl-1,2,3,6-tetrahydropyridine (MPTP), 6-hydroxydopamine (6-OHDA), or rotenone [12-17]. The intranigral or systemic administration of lipopolysaccharide (LPS), which is a gram-negative bacteriotoxin that activates microglial cells, can selectively initiate DA neuronal cell death [18,19], though LPS does not seem to have a direct harmful effect on neurons [20]. These events were attenuated by pretreatment with anti-inflammatory agents, such as naloxone [21] and minocycline [22]. Many epidemiological studies of PD indicate that anti-inflammatory medications have been identified as potentially effective in reducing the risk of PD [23-25]. These findings strongly suggest that microglial activation-derived oxidative stress and neuroinflammation could increase neurotoxicity and might play an important role in DA neuronal degeneration.

In this study, we characterized the activated microglia in SN following medial forebrain bundle (MFB) transection by using a rat MHC class II marker (OX6) and phagocytic marker (ED1), and furthermore, we demonstrated production of ROS from activated microglia using hydroethidine. After that, we examined whether administration of activated microglia could accelerate DA neuronal degeneration. In particular, we also showed that the inactivation of microglia with a microglia inhibitory factor (tuftsin fragment 1-3) could be neuroprotective by demonstrating a high survival rate of axotomized DA neurons in rats. Based on these findings, we indicated that activated microglia could have a deteriorative effect on axotomized DA neuronal survival and that well-controlled blockade of microglial activation might be neuroprotective.

## Methods

### Animal care and MFB transection

All experimental procedures performed on the animals were approved by the Animal Review Board of the Eulji University (EUIACUC-10-13) in accordance with the National Institutes of Health Guide for the Care and Use of Laboratory Animals (NIH Publication No. 80-23, revised 1996). All efforts were made to minimize the numbers of animals used and ensure minimal suffering of those animals. Adult male Wistar rats (n = 90, Charles River Lab, Wilmington, USA) weighing 250-300 g at the time of surgery were used for the following experiments: immunohistochemistry and hydroethidine histochemistry (n = 8), western blot assay (n = 12), isolation of microglia (n = 12), transplantation of microglia (n = 12), and administration of tuftsin fragment 1-3 (n = 46). Animals were housed in groups of two or three, with food and water in a room maintained at constant room temperature (20 ~ 22°C) with a 12:12-h light-dark cycle.

The MFB transection was carried out as previously described [26]. Animals were anesthetized by intraperitoneal injection of ketamine (70 mg/kg) and xylazine (8 mg/kg) mixture and secured in a stereotactic apparatus (Stoelting Co., Wood Dale, USA). After exposure of the skull, a small hole was made with a dental drill in the right side of the skull at coordinates 3.0 mm caudal to bregma and 2.8 mm right to midline. A cannula, housing a retractable wire knife (David Kopf Instruments, Tujunga, USA), was lowered through the hole to a depth of 9.0 mm from the dural surface and then the wire knife was extended 2.2 mm toward the midline, and the knife was slowly raised 3.0 mm and subsequently lowered back to its original position to ensure complete excision of the whole MFB. After surgery, animals were kept on a heating plate at 37°C until recovery was complete. The contralateral side of the brain was used as internal control.

### Tuftsin fragment 1-3 administration using mini-osmotic pump

Following unilateral MFB transection, under consecutive anesthesia, a cannula (Brain Infusion Kit II, Alzet; Durect Co., Cupertino, USA) which was connected to a mini-osmotic pump (Alzet) by a vinyl catheter tube was stereotactically implanted into the contralateral lateral ventricle (0.8 mm cranial to bregma, 1.5 mm left to midline, and 4.0 mm depth from dural surface). All infusions were performed using a 200 μl mini-osmotic pump (Model 2002 for 2 week-group and Model 2004 for 4 week-group animals). The pumps were designed to infuse continuously either tuftsin fragment 1-3 (30 ng/kg per day; Peptron, Daejeon, Korea) or vehicle solution (artificial CSF; CMA Microdialysis AB, Solna, Sweden) for 14 days or 28 days. Dosage of tuftsin fragment 1-3 was chosen in accordance with that of previous studies [27-29]. The connected osmotic pump was inserted subcutaneously in the back of the animal through the interscapular pocket. The scalp was sutured and then the animal was removed from the stereotactic frame and allowed to recover from surgery in a warm environment. The animals were carefully observed and monitored for several hours after recovery from anesthesia.

### Isolation and culture of microglia from adult rat midbrain

The isolation of microglial cells from adult rats was performed using methods previously described by Brewer and Torricelli [30], with some modifications. Following cardiac perfusion with normal saline to remove the peripheral blood cells, the rat brain was rapidly extracted and placed on a brain blocker (David Kopf Instruments), and sliced into about 5 mm coronal block including SN of which the position was determined according to the Paxinos and Watson [31]. The tissue block was cut through the arbitrary horizontal line crossing the inferior border of the both medial geniculate bodies, and then cut through the arbitrary vertical line dividing SN into right and left halves.

The isolated tissue blocks were dissected out in ice-cold HAGB [HA medium (Brain Bits, Springfield, USA) supplemented with 2% B-27 (Invitrogen, Carlsbad, USA) and 0.5 mm Glutamax (Invitrogen)]. They were chopped into pieces lesser than 0.5 mm^3^ using MacIlwain tissue chopper (The Mickle Lab., Guildford, UK) and digested for 30 min at 30°C with papain (30 units/ml; Worthington, Lakewood, USA) in calcium-free HA medium (Brain Bits) with 0.5 mm Glutamax. The digested tissues were transferred to a 15 ml tube containing 2 ml HABG and carefully triturated 15 times through a 5 ml plastic pipette. Samples were allowed to settle by gravity for about 2 min, and then the supernatant containing dissociated neurons and neuroglia was transferred to a fresh tube. Twice more, the sediment was suspended in 2 ml HABG, triturated 15 times, allowed to settle for 2 min and the supernatants combined until 6 ml of supernatant was collected. The collected supernatant was loaded upon the top of the prepared OptiPrep step gradient (Sigma-Aldrich Co., St. Louis, USA) solution. The OptiPrep step gradient was made in four 1 ml steps of 17.0, 13.5, 10.0, and 7.5% OptiPrep gradient in HABG. The cell suspension was centrifuged at 800 g for 15 min at room temperature and the pellet, which is enriched with microglia, was harvested.

For the transplantation of activated microglia, the pellet which was collected from SN treated with MFB transection 7 days ago was resuspended with HBSS (Gibco, Carlsbad, USA) and transplanted immediately. For immunofluorescence, the pellet was resuspended in 2 ml DMEM and 50,000 cells were plated on Ø 12-mm glass coverslips, which had previously been coated with poly-D-lysine (70–150 kDa, Sigma-Aldrich Co.) at 37°C overnight. After allowing cells to adhere for 2 h in 37°C incubator at 5% CO_2_, the coverslip was employed for immunofluorescence.

### Stereotactic intranigral transplantation of activated microglia

One week after unilateral MFB transection, the isolated microglia were transplanted into the operated (ipsilateral) side of SN. Rats were deeply anesthetized and placed in a stereotaxic frame with microinjector unit (David Kofp Instruments). Stereotactic coordinates for the injection were 5.0 mm caudal to bregma, 1.5 mm right to midline, and 7.0 mm from dural surface. A total of 1×10^5^ microglia suspended in 200 μl HBSS buffer was injected into the ipsilateral SN with a Hamilton syringe (22-gage). The injections were made at a rate of 100 μl/min and the needle was left in place for 2 min after each injection and slowly withdrawn. Sham operated animals were infused with the same volume of HBSS buffer alone. The rats were placed back into cages where they were allowed to recover and were sacrificed at 7 days post transplantation.

### Sample preparation and immunohistochemistry

At 7, 14, and 28 days after unilateral MFB transection, or 14 and 28 days following administration of tuftsin fragment 1-3, or 7 days after transplantation of activated microglia, the animals were deeply anesthetized. The animals were perfused transcardially with 100 ml of ice-cold 0.1 M sodium phosphate buffer (PB; pH 7.6) containing 0.5% sodium nitrite and 10 U/ml heparin, followed by 200 ml of 4% paraformaldehyde in 0.1 M PB. The brain was carefully removed from the skull and post-fixed for 12 h in the same fixative and infiltrated with 30% sucrose solution for 12-24 h at 4°C until they sank. The brain was then placed on a brain blocker (David Kopf Instruments) and sliced into 5 mm coronal block including the SN. The coronal blocks were rapidly frozen in 2-methylbutane chilled on dry ice and mounted in Tissue-Tek OCT compound (Sakura Finetechnical Co., Tokyo, Japan). Serial coronal sections with 40 μm-thickness were obtained on a Cryostat Microtome (Leica Microsystems Inc., Wetzlar, Germany).

Sections were washed for 10 min in 0.1 M PB containing 0.9% saline (PBS) and endogenous peroxidase activity was quenched by incubating the tissue sections with 0.3% hydrogen peroxide in PBS for 30 min. The sections were rinsed in 0.1 M PBS and incubated in 0.1 M PBS containing 5% normal goat serum and 0.1% Triton X-100 for 30 min to reduce non-specific staining. The sections were then incubated with primary antibodies, diluted in 0.1 M PBS containing 0.1% Triton X-100 (PBST) for 16 h at 4°C. Primary antibodies used in this study were as follows: rabbit anti-tyrosine hydroxylase (TH) monoclonal antibody (1:2000; Chemicon, Temecula, USA), mouse monoclonal anti-rat CD11b antibody (OX42; 1:500; Serotec, Oxford, UK), mouse monoclonal anti-rat MHC class II antibody (OX6; 1:500; Serotec), and mouse monoclonal anti-rat CD68 antibody (ED1; 1:500; Serotec). OX42 was used as a pan-microglial cell marker, OX6 and ED1 were used for identification of activated microglia, since it has been reported that the expression of ED1 is upregulated in microglia during phagocytosis [32] and the activated microglia express high level of MHC class II [3]. Sections were washed in PBST and incubated for 2 h with biotinylated goat anti-rabbit IgG (1:200; Vector Labs, Burlingame, USA) for TH or biotinylated goat anti-mouse IgG (1:200; Vector Labs) for OX42, OX6, and ED1. Following rinses, sections were exposed to avidin-biotin peroxidase complex (Vector Labs) which was applied for 1 h prior to incubation during 3-5 min for peroxidase detection using 3,3′-diaminobenzidine tetrahydrochloride (DAB; Sigma-Aldrich Co.). Light photomicrographic images were acquired on a Nikon Optiphot microscope (Nikon Inc., Tokyo, Japan) fitted with a Nikon digital camera (DXM1200; Nikon Inc.), using Nikon ACT-1 image capture software (version 2.2; Nikon Inc.).

### Immunocytofluorescence labeling and semi-quantitative analysis

Indirect immunofluorescent labeling with primary antibodies against CD11b (OX42; microglia), GFAP (astrocytes), and NeuN (neurons) were performed to assess the cell type and purity of the isolated microglia from adult rat brains. We also conducted OX6 and iNOS (inducible nitric oxide synthase) immunofluorescent labeling to investigate whether microglial cells isolated from ipsilateral SN were activated. Cells plated on 12-mm glass coverslips were fixed with 4% paraformaldehyde and 4% sucrose solution for 10 min. Following incubation in PBS containing 10% normal horse serum for 30 min, the cells were incubated overnight at 4°C with OX42 (1:100), GFAP (mouse monoclonal anti-GFAP antibody, 1:100, Chemicon), NeuN (mouse monoclonal anti-NeuN antibody, 1:100, Chemicon), OX6 (1:100), and iNOS (goat polyclonal anti-iNOS antibody, 1:100, Chemicon). After rinsing in PBS, a Cy3-conjugated horse anti-mouse secondary antibody (1:200; Jackson ImmunoResearch Lab., West Grove, USA) for OX42, GFAP, NeuN, and OX6 or Cy3-conjugated horse anti-goat secondary antibody (1:200; Jackson ImmunoResearch Lab.) for iNOS was applied for 2 h at room temperature. The coverslips were then incubated with Hoechst 33342 (Cell Signaling Technology Inc., Beverly, USA) to visualize the nuclei, and mounted upon slide glass with Vector-shield medium (Vector Labs).

For semi-quantitative analysis, the number of OX42, GFAP, NeuN, OX6, and iNOS-immunoreactive (-ir) cells and the total number of Hoechst positive nuclei were counted by two of the authors independently. Ten counting fields (at 200 magnifications; one counting field is 700 × 500 um) in each coverslip were selected randomly, and the images were captured with an Olympus Ax 70 microscope (Olympus Inc., Tokyo, Japan) fitted with a Carl Zeiss AxioCam MRC digital camera (Carl Zeiss Inc., Jena, Germany). The percentage was calculated by dividing the average number of OX42-ir, GFAP-ir, NeuN-ir, OX6-ir, and iNOS-ir cells by the average number of Hoechst positive nuclei. Three experimental animals were used. The statistical significance was assessed by one-way ANOVA followed by Dunnertt’s test. Values of *P* < 0.05 were considered significant.

### *In situ* detection of O_2_^-^and O_2_^-^-derived oxidant production and OX6/TH double immunofluorescence

*In situ* visualization of reactive oxygen species (ROS, O_2_^–^and O_2_^**-**^**-**derived oxidant) generated by microglia was assessed by hydroethidine (HEt) histochemistry [33,34]. At 7, 14, and 28 days after unilateral MFB transection, HEt (2 mg/kg in saline containing 1% dimethysulfoxide; Molecular Probes, Eugene, USA) was administered intraperitoneally. After 30 min, the brains were harvested as described above. Midbrain sections mounted onto gelatin-coated glass slides were examined for oxidized product of hydroethidine, ethidium accumulation, by fluorescence microscopy (excitation, 510 nm; emission, 580 nm).

OX6/TH double immunofluorescence labeling was performed at HEt treated sections to examine whether activated microglia produce ROS. Sections were incubated with PBS containing 10% normal goat serum for 30 min, followed by the application of the mixture of TH (1:200) and OX6 (1:50) antibodies for 16 h at 4°C. After rinsing in PBS, a mixture of AMCA-conjugated donkey anti-rabbit secondary antibody (1:200, Jackson ImmunoResearch Lab.) and FITC-conjugated donkey anti-mouse secondary antibody (1:200, Jackson ImmunoResearch Lab.) was applied for 2 h at room temperature. All procedures were conducted in a dark condition.

### Stereological cell count of TH-immunoreactive DA neurons

Stereological unbiased procedures using the optical dissector method were carried out to provide the percentages of TH-immunoreactive (TH-ir) neurons in the SN, as previously described [26]. Following every fourth section through the entire SN (levels: AP-4.5 mm to-6.3 mm to bregma) was sampled into three series of sections, TH immunohistochemistry was performed as described above. The borders of the SN at all levels were defined to Kirik’s method [35]. StereoInvestigator software (MicroBrightField Inc., Williston, USA) was used to delineate the one side of SN at 4× objectives and generate 20% counting area. A counting frame (1600 μm^2^) was placed randomly and systematically moved through all counting areas. Actual counting was done under 630 magnifications. The sampling volume in the Z-axis extended 25 μm deep after excluding 3 μm from both top and bottom surfaces. The total number of the ipsilateral or contralateral TH-ir neurons was obtained by summing the profiles of each section and the average number was estimated.

To examine the survival rate of TH-ir neurons following administration of tuftsin fragment 1-3 or transplantation of activated microglia, the survival percentages of TH-ir neurons in the ipsilateral SN were calculated by dividing the average number of the ipsilateral TH-ir neurons by the contralateral ones. Five to seven animals at each time point were used.

### Western blot analysis

The midbrain tissue blocks including the ipsilateral or the contralateral SN were obtained as described above and homogenized in a protein extraction solution (Pro-prep protein extraction solution; Intron Biotech, Seoul, Korea) using polytron homogenizer. Protein concentrations were determined using a Bio-Rad colorimetric protein assay kit following the manufacture’s guide (Bio-Rad, Hercules, USA). Equal amount (20 mg) of protein were loaded and separated by sodium dodecyl sulfate (SDS)-polyacrylamide gel electrophoresis, and transferred onto a nitrocellulose membrane (Invitrogen). Membranes were blocked with 5% nonfat milk for 1 h and incubated overnight at 4°C with TH (1:5000), OX42 (1:2000), OX6 (1:2000), ED1 (1:2000), and mouse monoclonal anti-beta-actin (1:5000, Chemicon) antibodies. Horseradish peroxidase-conjugated antibody (1:500; Amersham Pharmacia Biotech, Piscataway, USA) was used as a secondary antibody. Band detection was performed using the enhanced chemiluminescence (ECL) detection system (Amersham Pharmacia Biotech). For semi-quantitative analyses, the densities of each band on immunoblots were normalized against that of beta actin by using the Image J software.

## Results

### MFB transection induced gradual loss of DA neurons and microglial activation

**Figure 1:**
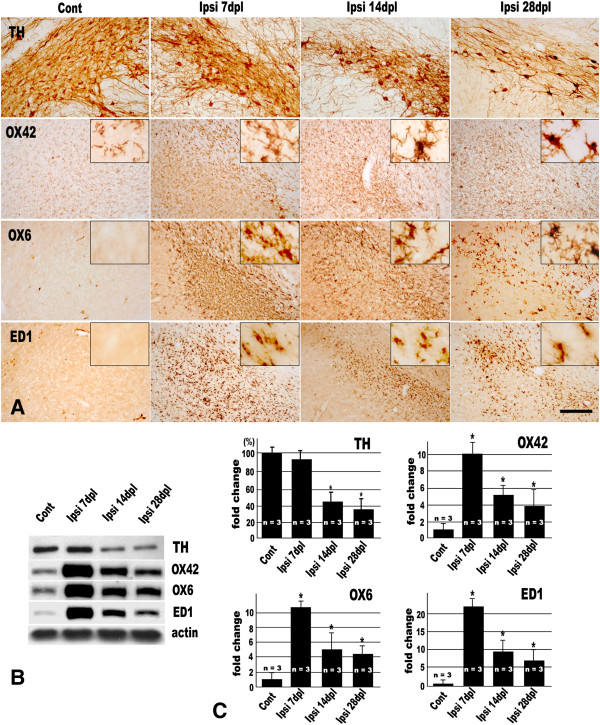
**MFB transection induces significant loss of TH-ir neurons and microglial activation in the ipsilateral SN. (A)** TH, OX42, OX6, and ED1 immunohistochemistry in the contralateral (Cont) and ipsilateral (Ipsl) SN at 7, 14, and 28 days post-lesion (dpl) of unilateral MFB transection. Insets in **(A)** represent higher magnification of each photograph. Significant reduction in the number of TH-ir neurons is noted in the ipsilateral SN at 14 and 28 dpl. The resting ramified microglia showing weak OX42 immunoreactivity are distributed evenly in the contralateral SN and they are not immunolabeled with ED1 or OX6. However, in the ipsilateral SN, quite numerous activated microglia with increased OX42 immunoreactivity are found and they are also immunoreactive with OX6 and ED1. The activated microglia retain enlarged soma and shorter, thicken cytoplasmic processes. Scale bar represents 100 μm. **(B)** Changes in expression level of TH, OX42, OX6, and ED1 by western blot assay. **(C)** Quantification of results from western blot assay in **(B)**. The density of each TH, OX42, OX6, and ED1 band was normalized against that of beta-actin. Values were normalized to control and expressed as the mean ± S.E.M. (*n* = 3). *P < 0.05 compared with value from control (ANOVA with *post hoc* Student’s *t* test).

Compared with the contralateral SN, a time-dependent decrease of the number of the TH-ir DA neurons was noted in the ipsilateral SN (Figure 1A). When the amount of the TH protein in the ipsilateral SN was measured by western blot assay, the percentage of that to the contralateral counterpart was 94.4 ± 6.7% (mean ± SD), 43.8 ± 10.5%, and 36.6 ± 9.9% at 7, 14, 28 days post-lesion (dpl), respectively (Figure 1B, C).

The progressive loss of DA neurons was accompanied by microglial activation. In the contralateral SN, resting microglia characterized by small cell bodies and long, slender processes with weak OX42 immunoreactivity were distributed throughout SN. There were little or no detection of OX6 or ED1-ir cells in the contralateral SN parenchyma, except for a few OX6-ir or ED1-ir cells around the blood vessels which were believed to be perivascular cells. However, a considerable number of activated microglia retaining enlarged soma and shorter, thicken cytoplasmic processes were crowded in the ipsilateral SN after unilateral MFB transection. They showed intense OX42, OX6, and ED1 immunoreactivity (Figure 1A). To confirm the results of the immunohistochemistry, we examined the changes of the protein level of CD11b (OX42), MHC class II (OX6), and CD68 (ED1) by western blot assay. Quantification of the bands by densitometry revealed that the expression levels of CD11b (OX42), MHC class II (OX6), and CD68 (ED1) in the ipsilateral SN were above 5 folds higher than that of the contralateral SN (Figure 1B, C).

### OX6-positive activated microglia produce superoxide

**Figure 2:**
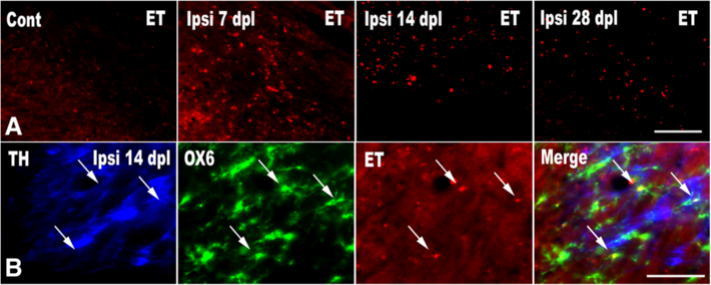
**The activated microglia produce ROS.** Hydroethidine (2 mg/kg), which can be converted to ethidium by ROS, was injected to the experimental animals intraperitoneally. After 30 min, brains were harvested and sections of SN were prepared for hydroethidine histochemistry to detect superoxide. **(A)** Representative ethidium fluorescence of the contralateral (Cont) and ipsilateral (Ipsi) SN at 7, 14, and 28 dpl following unilateral MFB transection. The number of ethidium fluorescent spots increases to peak level at 7 dpl, and then decreases gradually by 28 dpl. **(B)** Immunofluorescent images of TH, OX6, ethidium (ET), and their merged image in the ipsilateral SN at 14 dpl. Many OX6 immunoreactive microglia are co-localized with ethidium (arrows). Scale bars represent 50 μm.

Recent accumulating *in vivo* and *in vitro* evidence suggests that activated microglia produce ROS (O_2_^-^and O_2_^**-**^**-**derived oxidant) [36]. To investigate whether ROS production has changed with the progress of neurodegeneration in the ipsilateral SN, we performed *in situ* visualization of ROS (O_2_^-^and O_2_^**-**^**-**derived oxidant) with fluorescence of ethidium, which is oxidized hydroethidine *in vivo*. The fluorescent products of oxidized hydroethidine (i.e., ethidium accumulation) were significantly increased at 7 dpl in the ipsilateral SN and decreased gradually by 28 dpl (Figure 2A). The distribution of ethidium fluorescent was correctly matched with that of activated (OX6-ir or ED1-ir) microglia.

Later, we conducted TH/OX6 double immunofluorescence labeling to detect what kind of cells produce ROS, as well as to confirm the spatial relationship between activated microglia and TH-ir DA neurons. The results showed that numerous ethidium fluorescent nuclei were embedded within the cell body of OX6-ir microglia (arrows, Figure 2B). Furthermore, these activated microglia retaining ethidium fluorescence were closely besieged with TH-ir DA neurons (Figure 2B).

### Transplantation of activated microglia exacerbates the degeneration of axotomized DA neurons

**Figure 3:**
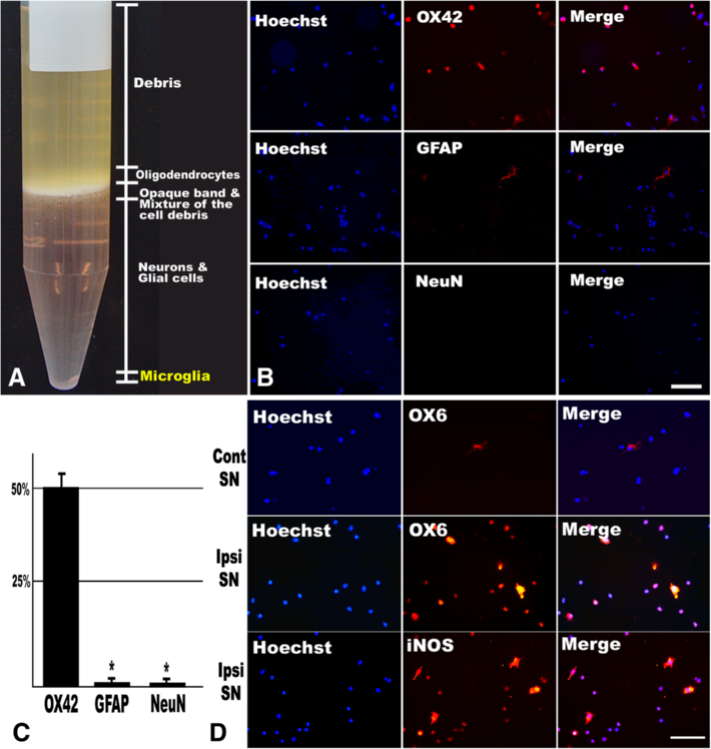
**Isolation of microglia from adult rat mesencephalon using density gradient fractionation. (A)** Cellular pool from the midbrain of 10-week-old Wistar rat using OptiPrep step gradient separation method. The fraction and cellular designation were based on the previous study [30]. Note the pellet at the bottom that contains microglia. **(B)** Double immunocytofluorescence labeling of Hoechst (nucleus) and one of the microglial (OX42), astrocytic (GFAP), and neuronal (NeuN) markers using primary cultured cells obtained from the pellet in **(A)**. Scale bar represents 100 μm. **(C)** Semi-quantification of the number of OX42-ir, GFAP-ir, and NeuN-ir cells against Hoechst positive nuclei. The results are expressed as the percentage of the number of OX42-ir, GFAP-ir, and NeuN-ir cells to the total number of Hoechst-ir nuclei. *P < 0.05 compared with value from OX42-ir result (ANOVA with *post hoc* Student’s *t* test). **(D)** Immunocytofluorescence of OX6 and iNOS in primary cultured microglia originated from the contralateral (Cont) or ipsilateral (Ipsi) SN at 7 dpl. Microglia cultivated from the ipsilateral SN are highly immunoreactive with OX6 and iNOS, meaning that they are highly activated and produce high level of reactive nitrogen species. Scale bar represents 20 μm.

After density gradient centrifugation, a distinct opaque band and a small pellet could be obtained (Figure 3A). According to a previous report [30], the opaque band and its upper fraction is enriched with oligodendrocytes and cell debris. There is enrichment for neurons from the lower edge of the dense band to 0.5 ml from bottom of the tube and the loose pellet on the bottom contains microglia. Therefore, in this study, only the pellet fraction was collected and used. Cell yields reached 1.28 × 10^6^ cells per 1 mg of SN tissue block. Immunofluorescent labeling of the isolated pellet indicated a highly homogeneous population of very small and round cells, which were uniformly stained with microglial marker (OX42; ≥ 51%), but were negative with the astroglial (GFAP; ≤ 0.5%) or neuronal markers (NeuN; ≤ 0.3%) (Figure 3B, C).

To confirm whether the mesencephalic microglia isolated from the ipsilateral SN have retained the characteristics of activated microglia, immunofluorescent staining of OX6 and iNOS was conducted. A great number of microglia prepared from the ipsilateral SN at 7 dpl after MFB transection were highly immunoreactive with OX6 and iNOS. The percentage of OX6-ir cells/Hoechst-ir cells is 14.3 ± 2.6% and the percentage of iNOS-ir cells/Hoechst-ir is 18.8 ± 3.2%. Some of them showed typical morphologic characteristics of activated microglia, with large soma and thickened processes. However, microglia isolated from the contralateral SN displayed OX6-negative immunophenotype (Figure 3D).

**Figure 4:**
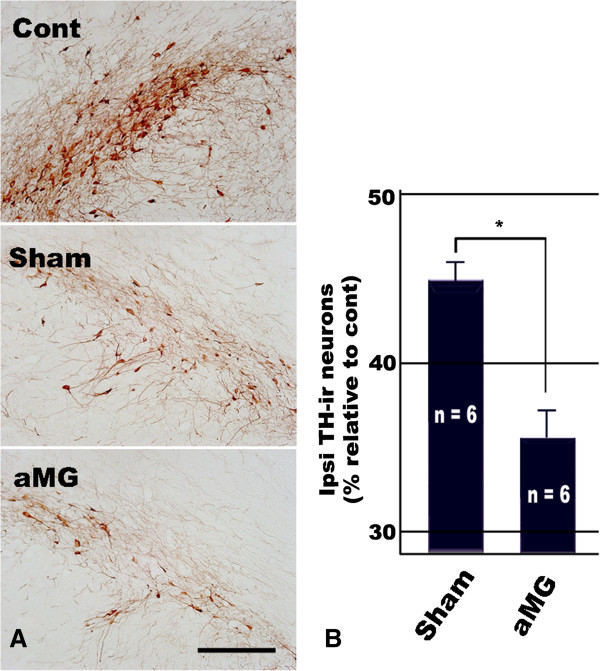
**Exogenous activated microglial transplantation into the ipsilateral SN accelerated the DA neuronal cell death. (A)** TH immunohistochemistry in the contralateral (Cont) and ipsilateral SN at 7 days after vehicle (Sham) or activated microglia (aMG) implantation. Scale bar represents 300 μm. **(B)** Graph representing the relative percentage of the number of nigral TH-ir DA neurons. For quantitative analysis, every fourth section among 40 μm serial brain sections was immunohistochemically stained with antibodies against TH, and the number of TH-ir neurons were counted using a stereological technique in the whole SN. The percentage was analyzed by dividing the total number of TH-ir neurons in the ipsilateral SN by that of the contralateral SN. Significant reduction in the number of SN TH-ir neurons of the aMG group, compared with that of the vehicle treated group, indicates that transplantation of exogenous activated microglia into the ipsilateral SN accelerates the axotomized DA neuronal cell death. Values were expressed as the mean ± S.E.M. (*n* = 6). *P < 0.05 compared with value from sham control (ANOVA with *post hoc* Student’s *t* test).

When compared with the sham control, a clear reduction of TH-ir cell number/density was obvious in the ipsilateral SN where the activated microglia had been transplanted (Figure 4A). Subsequent unbiased stereological analysis confirmed that the proportion of the TH-ir neurons in the ipsilateral SN of activated microglia transplanted groups was 36.4 ± 3.2% and that of sham control groups was 45.1 ± 2.3% (Figure 4B).

### Inhibition of microglial activation attenuates DA neuronal cell loss

Tuftsin fragment 1-3 treated animals were monitored for overall health, weight loss, and activity during the course of drug infusion. There were no remarkable physical or behavioral changes compared with the vehicle-treated or un-operated animals.

**Figure 5:**
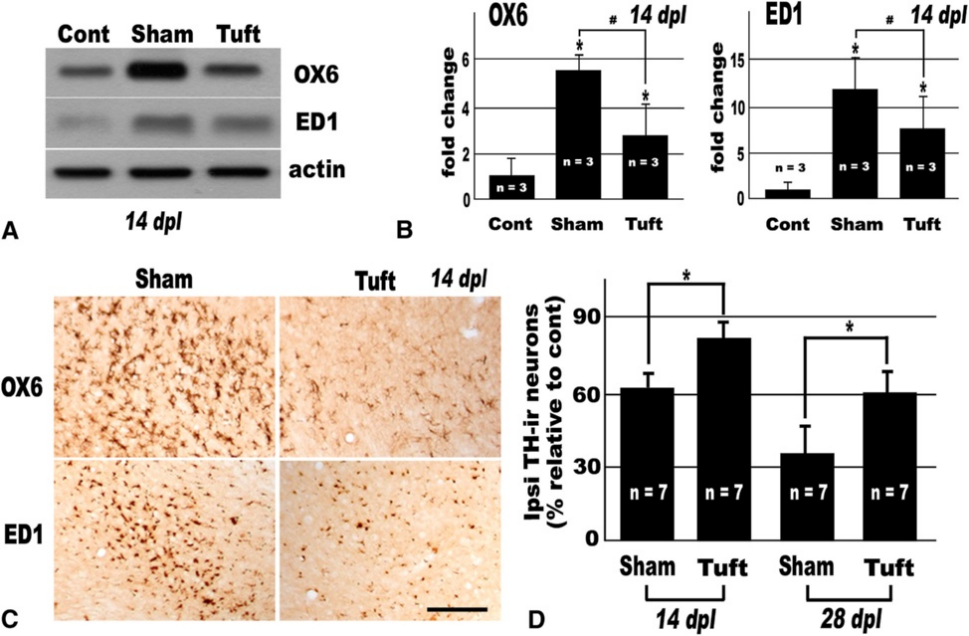
**Tuftsin fragment 1**-**3 decreased microglial activation and increased the survival rate of DA neurons.** Tuftsin fragment 1-3 or artificial CSF was administrated into the contralateral lateral ventricle using mini-osmotic pump for 14 and 28 days. **(A)** Western blot assay of OX6 and ED1 in the ipsilateral SN at 14 dpl following unilateral MFB transection and administration of tuftsin fragment 1-3 or artificial CSF. **(B)** Quantification of results from western blot assay in **(A)**. The density of each OX6 and ED1 band was normalized against that of beta-actin. Values were normalized to control and expressed as the mean ± S.E.M. (*n* = 3). *P < 0.05 compared with value from control and ^#^*P* < 0.05 compared with value from sham control (ANOVA with *post hoc* Student’s *t* test). **(C)** OX6 and ED1 immunohistochemistry in the ipsilateral SN at 14 dpl following unilateral MFB transection. In vehicle treated animals (Sham), innumerable OX6-ir and ED1-ir microglia are crowed in the ipsilateral SN. However, in tuftsin fragment 1-3 treated animals (Tuft), the OX6 and ED1 immunoreactivities are deeply suppressed. Scale bar represents 250 μm. **(D)** Graph representing the relative percentage of the number of nigral TH-ir DA neurons. For quantitative analysis, every fourth section among 40 μm serial brain sections was immunohistochemically stained with antibodies against TH, and the number of TH-ir neurons were counted using a stereological technique in the whole SN. The percentage was analyzed by dividing the total number of TH-ir neurons in the ipsilateral SN by that of the contralateral SN. Values expressed as the mean ± S.E.M. (*n* = 7). *P < 0.05 compared with value from sham control (ANOVA with *post hoc* Student’s *t* test).

Tuftsin fragment 1-3 administration decreased the MHC class II (OX6) and CD68 (ED1) expression level above 2 folds lower than that of the vehicle treated group at 14 dpl (Figure 5A, B) and showed dramatic decrease in intensities of OX6 and ED1 immunoreactivities (Figure 5C). In accordance with suppression of microglial activation, the DA neuronal cell loss was attenuated by administration of tuftsin fragment 1-3. In the vehicle administrated group, the proportions of TH-ir neurons in the ipsilateral SN were 61.0 ± 4.0% at 14 dpl and 35.2 ± 2.6% at 28 dpl. On the other hand, the proportions of that in the tuftsin fragment 1-3 treated groups were 80.8 ± 4.8% at 14 dpl and 60.3 ± 5.1% at 28 dpl (Figure 5D).

## Discussion

Notwithstanding employment of some genetically based models of PD in rodents and invertebrates, two main experimental animal models of PD have been widely used: (1) the selective degeneration of DA neurons with the neurotoxins, such as N-methyl-4-phenyl-1,2,3,6-tetrahydropyridine (MPTP, [37,38]) or 6-hydroxydopamine (6-OHDA, [39]) and (2) the induction of degenerative changes of DA neurons through precise transection of the nigrostriatal afferent fibers [40]. The toxicity of MPTP and 6-OHDA is believed to result from inhibition of complex I of the mitochondrial electron transport chain [41] and could lead to acute and almost complete DA neuronal cell death within a short time (1-3 days). However, the transection of the adult rat MFB leads to different degrees and rates of DA neuronal death. Our previous studies showed that a time-dependent, progressive loss of DA neurons is the main characteristic of the MFB transection model and this surgical approach permitted axotomized neurons to live for a significant length of time *in situ*; the statistically significant decrease of DA neurons was observed from 7 days post-transection and about 40% of axotomized DA neurons survived till 70 days after MFB transection [26,42]. Therefore, MFB transection model is more suitable to study the relationship between DA neuronal degeneration and the surrounding environmental effects, such as neuroglial function.

At 7 days after MFB transection, the protein level of TH in the ipsilateral midbrain tended to decrease compared to the contralateral side, but this decrease was not significant by western blot assay (Figure 1). This result was not consistent with our previous reports: at this time point, when measured by unbiased stereological method, the number of TH-ir neurons in the ipsilateral SN decreased significantly about 10% [26,42]. This discrepancy could be explained by the difference in methods used. In contrast to the stereological method which confined the region of interest to the SN, in western blot assay, the used tissue block included the ventral tegmental area (VTA) as well as SN. Therefore, the TH protein in the VTA might be summed up in western blot assay and could mask the decrease of TH protein level in the SN. The possibility also cannot be ruled out that the remaining DA neurons in the SN produce more TH protein to compensate the loss of neighboring DA neurons. By and large, the total amount of TH protein was decreased as time elapsed, which means that progressive DA neuronal death occurred following MFB transection.

The precise mechanism of axotomized DA neuronal death is poorly understood, and it might be different from other parkinsonian animal models, because MPTP associated death is TUNEL-positive [43,44], while MFB transection-induced cell death is TUNEL-negative [45,46]. Therefore, some authors argued that the cell death mechanism of axotomized DA neurons is entirely necrotic [45,46]. However, we have presented some different opinions that the mechanism of nigral cell death after axotomy is another type of apoptosis, such as apoptosis-like caspase-independent apoptosis [26,47]. The latter possibility is suggested by the findings that DAPI staining showed apoptocic figures (i.e. chromatin clumps) in axotomized DA neurons [47]. And many TH-ir neurons, undergoing various steps of consecutive neurodegenerative changes in the ipsilateral SN, retained phosphorylated c-Jun and activating transcription factor 3 in the condensed or fragmented nuclei [26]. It is widely accepted idea that c-Jun phosphorylation triggers the cascades of programed cell death in the injured dopaminergic neurons [48].

As the gradual DA neuronal death went on, to clear the resultant cell debris and dead or dying neurons from the CNS parenchyma, microglial activation was noted in the ipsilateral SN. Morphologically activated microglia (with large soma as well as shorten and thicken processes) were crowded and they actively expressed MHC class II (OX6-ir) and showed phagocytic characteristics (ED1-ir) (Figure 1). Moreover, these activated microglia produced ROS (O_2_^-^and O_2_^**-**^**-**derived oxidant) and showed a close spatial relationship with collateral TH-ir DA neurons (Figure 2). In this study, hydroethidine (also known as dihydroethidium) was adopted to detect ROS production *in situ*. Hydroethidine is easily taken up by living cells and shows blue fluorescence. Within the living cell, hydroethidine can be directly oxidized in the presence of ROS, such as O_2_^-^, and then, it is converted to red fluorescent ethidium, which in turn is trapped in the cells by intercalation into DNA [32,49].

Microglial activation involves characteristic morphological transformation from resting to the activated state, and activated microglia release ROS as well as various proinflammatory and neurotoxic factors such as tumor necrosis factor-alpha, interleukin-1beta and so on. [2-5,50]. The ROS can cross cell membranes easily and induce neuronal death by causing oxidative damage to cellular components [51]. Actually, activated microglia in culture secrete large amounts of H_2_O_2_ and NO in a process known as 'respiratory burst’, and can directly damage cells and lead to neuronal cell death [52]. The potential clinical significance of this process has also been highlighted by a postmortem study of PD patients, which showed evidence of accumulation of activated microglia and oxidative modifications of proteins in the SN [53]. It is also well known that DA neurons in the SN possess reduced antioxidant capacity, such as accumulation of total iron and low intracellular glutathione level [54,55], which render DA neurons more vulnerable to oxidative stress, relative to other cell types. Moreover, the number of microglia is higher within the mesencephalon compared with other brain areas [56]. Taken together, these findings strongly suggest that the release of ROS from activated microglia could increase neurotoxicity to adjacent DA neurons and might contribute to neurodegeneration.

To confirm the role of activated microglia in DA neuronal degeneration, we implanted activated microglia into the SN treated with MFB transection and elucidate whether DA neuronal loss is accelerated. The most popularly used microglial cell line is BV-2 but its application is limited. For instance, BV-2 cells do not give reliable results in chemokine expression and release following LPS treatment compared with primary microglial cells. Moreover, it is natural that microglial cell line cannot help having different biological characteristics since its genetic immortalization might significantly affect its biology [57,58]. Therefore, in this study, we isolated adult primary microglia from rat brain 7 days after MFB transection.

Previous microglia isolation methodologies generally utilize whole brain regions. However, to utilize whole brain seems to be inadequate. The brain microenvironment is extremely dynamic depending on its distinct anatomical regions and the local neurochemical milieu [59]. Accordingly, the microglial phenotype and activation state, especially under pathological conditions, also have no choice but to show heterogeneity and brain region specificity [60]. The phenotypic diversity displayed by CNS microglia also reflects a functional diversity [61]. Thus, to isolate microglia within interesting brain anatomical loci is thought to be important to elucidate the exact role of microglia *in vivo*. Therefore, in this study, we isolated activated microglia from the adult rat midbrain of which MFB had been transected 7 days before, and implanted the activated microglia into the ipsilateral SN of the other rat directly of which MFB also had been transected 7 days ago. To isolate microglia from the SN region, we adapted Brewer and Torricelli’s protocols which had been developed to isolate and culture adult rodent neurons [30]. We could isolate highly enriched, quiescent microglia from adult rat SN. Other CNS cell types such as astrocytes and neurons were almost completely excluded (Figure 3). However, the exact identity of Hoechst-positive spots which were not stained with markers used in this study (OX42, NeuN, and GFAP) was obscure. It may be that some nuclei of microglia were not properly stained with OX42. Alternatively, it is possible that the nuclear debris was merely detected with Hoechst stain. In contrast to quiescent microglia isolated from normal rat SN, microglia isolated from the ipsilateral SN showed functionally activated phenotype possessing strong MHC class II and iNOS immunoreactivity (Figure 3). These results mean that the isolation procedure may not alter the microglial immunophenotypes.

We directly transplanted the activated microglia into the ipsilateral SN. So, the mechanical damage caused by the inserted syringe may induce the additional innate microglial activation. As a result, the ipsilateral SN, where the activated microglia were transplanted and treated with MFB transection 7 days ago, has three types of activated microglial cells. The first is innate activated microglia induced by MFB transection. The second is exogenous activated microglia transplanted from other brains. And the third is innate activated microglia induced by syringe insertion. The effect of the first and the third can be exclusory, because the sham controls had been also received the same surgical procedures. Therefore, we could assess the effect of exogenous activated microglia only, and concluded that the transplantation of exogenous activated microglia accelerates the axotomized DA neuronal death significantly (Figure 4).

Tuftsin fragment 1-3, a human IgG derived tripeptide, Thr-Lys-Pro, was first identified by Auriault and colleagues [62]. Since its potential role as a macrophage inhibitory factor was demonstrated, tuftsin fragment 1-3 has also been used as a microglia inhibitory factor in other studies, although its precise mechanism is unknown [28,63,64]. For example, Thanos and colleagues demonstrated that a single intravitreal injection of tuftsin fragment 1-3 inhibited phagocytic activity of microglia and increased the number of regenerating axons of retinal ganglionic cells by two to threefold [63]. Tuftsin fragment 1-3 also decreased ROS production from activated microglia, resulting in a promising reduction of brain edema and tissue damage and attenuation of functional deficits in ischemic animal brains [29].

In this study, we used tuftsin fragment 1-3 as a microglial inhibitory factor to investigate the role of activated microglia in the pathogenesis of axotomized DA neuronal degeneration. In tuftsin fragment 1-3 treated animal, the number and intensity of OX6-ir and ED1-ir activated microglial cells were dramatically decreased and the survival rate of ipsilateral DA neurons was improved about 20% compared with vehicle treated ones (Figure 5). As a consequence, tuftsin fragment 1-3 was proved to be an effective inhibitor of microglial activation and this inhibitory effect was thought to contribute to the increased survival rate of axotomized DA neurons.

## Conclusion

There is much evidence that brain inflammation plays a role in DA neuronal death in PD, although it is not known whether it is primary or secondary, and microglia are the main CNS cellular components which trigger and modulate CNS neuroinflammation. In the present report, we presented precise experimental evidences indicating that highly activated microglia could lead to neurotoxicity to axotomized DA neurons by producing ROS extensively. Therefore, a well-controlled blockade of microglial activation might be neuroprotective in some neuropathological conditions, if the neurons are potentially recoverable by themselves or by effective neuroprotectants.

## Competing interests

The authors declare that they have no competing interests.

## Authors’ contributions

BC and TB conceived and designed the experiments and helped to draft the manuscript. DS participated in the study design, and participated in all of the experiments as a first author. JH and HY conducted the animal experiments. DS and CP carried out immunohistochemistry, immunofluorescence, and stereologicial cell count. JL (4th author) and JL (5th author) participated in the western blot experiments. BP and RW carried out primary microglial cell culture and statistical analyses. All authors read and approved the final manuscript.
